# Splitting test experimental dataset of hollow concrete blocks

**DOI:** 10.1016/j.dib.2021.107646

**Published:** 2021-11-27

**Authors:** José Álvarez-Pérez, Milena Mesa-Lavista, Jorge H. Chávez-Gómez, Diego Cavazos-de-Lira, Bernardo T. Terán-Torres

**Affiliations:** Universidad Autónoma de Nuevo León (UANL), Facultad de Ingeniería Civil (FIC), Departamento de Estructuras, Av. Universidad, s/n CP. 66455, San Nicolás de los Garza, Nuevo León, México

**Keywords:** Hollow concrete blocks, masonry, indirect tensile strength, splitting test

## Abstract

Masonry structures are widely used nowadays for their advantages like low-cost workmanship, efficiency and fast construction techniques. The compressive strength of the materials that compose masonry (block and mortar) is very important to the behavior of the system, but the tensile strength is even more significant for the standards and building codes. In this work, a dataset for indirect tensile tests of hollow concrete blocks is obtained. Splitting tests as described in ASTM C-1006-13 are applied. Two sets of blocks were tested, one with medium compressive strength and the other with high compressive strength. The first set was tested in three directions named A, B, and C; the second one was tested in two directions, A and B. The data was collected with a servo-hydraulic machine. The data is presented in tables and can be used by material researchers, as well as in numerical modeling.

## Specifications Table

**Table:** 

Subject	Civil and Structural Engineering
Specific subject area	Hollow concrete blocks masonry
Type of data	Table, Image and Figure
How data were acquired	The tests were carried out in a servo-hydraulic machine
Data format	Raw and primary processed data
Parameters for data collection	The physical and mechanical parameters of blocks were obtained by measurement with caliper and the servo-hydraulic machine
Description of data collection	Splitting tests were performed according to the procedures established in the ASTM C-1006-13 standard [1]. Tests were displacement-controlled at a velocity of4000N/mm. This test method produces a line load along the bed surface of the masonry unit. The compressive load *P*, applied by bearing rods to the unit, results in tensile stresses distributed over the height of the unit *H*, in the split length of the unit *L*. The splitting tensile strength $\left(f_{ti}\right)$ of the specimen is then calculated by using an equation. This test method can be conducted with the rod oriented in either the longitudinal or the transversal direction to the bed surface. Data reported belongs to two sets of blocks, with medium and high compressive strength.
Data source location	Institution: Universidad Autónoma de Nuevo León, Instituto de Ingeniería Civil. City: San Nicolás de los Garza Town: Ciudad Universitaria Region: Nuevo León Country: Mexico (25°44′00.07″ N, 100°18′22.55″ W)
Data accessibility	Repository name: Mendeley Data Data identification number: Version 2, published in Mendeley Data, 2021, DOI:10.17632/53jc9d5wcz.2 Direct URL to data: https://data.mendeley.com/datasets/53jc9d5wcz/2
Related research article	Álvarez-Pérez, J. Mesa-Lavista, M. Chávez-Gómez, J.H. Fajardo-San Miguel, G., Experimental investigation on tensile strength of hollow concrete blocks, Materials and Structures, 54: 164 (2021). http://doi.org/10.1617/s11527-021-01761-3

## Value of the Data

• Dataset shows the obtained values of hollow concrete blocks that have been tested under indirect tensile tests, applying the ASTM C-1006-13 standard. The dataset can be used in the investigations of the characteristics of the materials.
• The data provided can be useful for researchers studying the strength of hollow concrete blocks masonry, and for its comparison with results from compressive strength or direct tensile tests.
• The data can be used in finite element modeling as part of the constitutive model.

## 1 Data Description

**Fig. 1:**
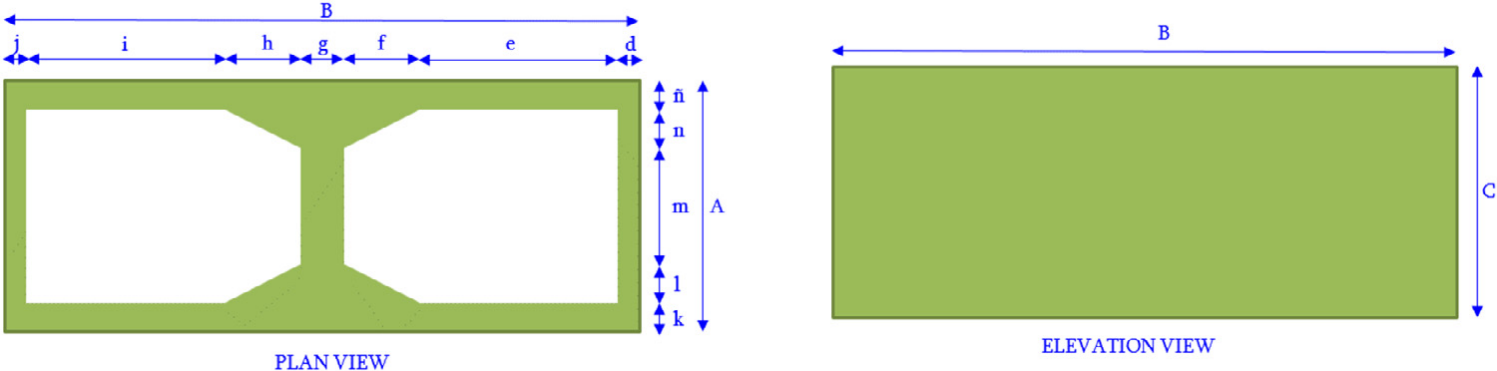
Dimensions of the block with medium compressive strength.

The collected data in this document is of two sets of hollow concrete blocks (HCB). The first, with medium compressive strength ($f{{}^{\prime }}_{C_{n}}=11.62\;\text{MPa}\;$over the net area), and the second set with high compressive strength ($f{{}^{\prime }}_{C_{n}}=28.8\;\text{MPa}\;$over the net area) [2]. The 1^st^ spreadsheet form the dataset shows all the dimensions measured from the 90 tested blocks, and the strength obtained for each specimen of the first set. The measurements and results of the second set are shown in the 2^nd^ spreadsheet, where 60 blocks were tested. The HCB, manufactured in the State of Nuevo Leon, Mexico, is of two cells and with cement and aggregate up to a size less than 10 mm. The nominal dimensions for the first set are 395 mm × 194 mm × 144 mm (length × height × thickness), while for the second set are 397 mm × 197 mm × 147 mm (length × height × thickness), which have a net to gross area ratio of about 0.57 and 0.55, respectively. Fig. 1 shows the plan and elevation view of the HCB, and the nomenclature of each block with medium compressive strength. The dimensions of the tested blocks with medium and high compressive strength are shown in https://data.mendeley.com/datasets/53jc9d5wcz/2.

**Fig. 2:**
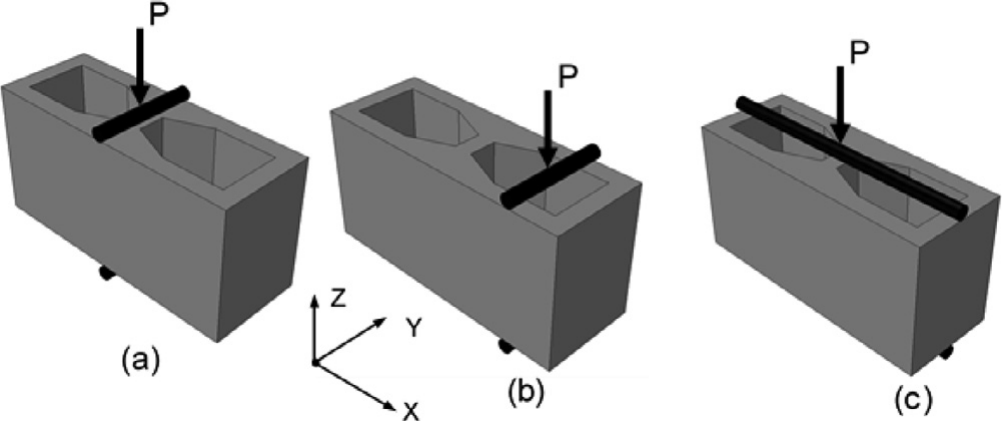
Splitting method. (a) In the x-direction over the gross area (Case A), (b) In the x-direction over the net area (Case C) (c) In the y-direction over the net area (Case B).

Splitting tests (Fig. 2) were performed according to the procedures established in the ASTM C-1006-13 standard [1]. The blocks with medium compressive strength were tested in the direction A, B, and C (Fig. 2), while the blocks with high compressive strength were tested in the A and B directions (Fig. 2a, and c).

Tests were displacement-controlled at a velocity of 4000N/mm. The splitting test has its origins in the works of [3]. This test method produces a line load along the bed surface of the masonry unit. The compressive load *P*, applied by bearing rods to the unit, results in tensile stresses distributed over the height of the unit *H*, in the split length of the unit *L*. The splitting tensile strength $\left(f_{ti}\right)$ of the specimen is then calculated by using the equation (1) [1].

(1) $$f_{t}=\frac{2P}{\pi LH}$$

Where: $f_{t}$ is the splitting tensile strength (MPa), *P* is the maximum applied load (N), *L* is the split length (mm) and, *H* is the distance between the rods (mm).

This test method can be conducted with the rod oriented in either the longitudinal or the transversal direction to the bed surface. Table 1, shows the summary of the dataset obtained from the splitting tests for blocks with medium compressive strength (1^st^ spreadsheet from the dataset).

**Table 1:** Summary of the tests for A, B and C specimens, with medium compressive strength.

A Specimens				B Specimens				C Specimens			
Max Load (N)	L (mm)	H (mm)	f_t_ (MPa)	Max Load (N)	L (mm)	H (mm)	f_t_ (MPa)	Max Load (N)	L (mm)	H (mm)	f_t_ (MPa)
34172.60	145.00	195.00	0.77	16179.80	78.29	194.60	0.68	19868.60	56.82	196.00	1.14
34535.20	145.00	195.00	0.78	8065.40	82.25	195.00	0.32	21432.60	53.19	195.00	1.32
42865.20	145.00	195.00	0.97	15444.80	77.83	194.30	0.65	17179.40	55.24	196.00	1.01
41375.60	145.00	195.00	0.93	13818.00	82.57	194.00	0.55	18914.00	56.00	195.00	1.10
48451.20	145.00	197.00	1.08	15680.00	81.65	195.00	0.63	17787.00	54.98	196.00	1.05
46226.60	145.00	195.00	1.04	16963.80	81.82	194.00	0.68	25558.40	53.35	195.00	1.56
37553.60	145.00	195.00	0.85	15797.60	78.56	196.00	0.65	23461.20	53.02	197.00	1.43
38090.50	145.00	195.00	0.86	18258.90	81.09	195.20	0.73	19891.40	54.12	196.00	1.19
38225.70	146.00	195.00	0.85	14212.60	81.16	194.90	0.57	17049.90	53.58	195.00	1.04
44297.50	145.00	195.00	1.00	19243.20	78.98	193.60	0.80	21460.20	55.93	196.00	1.25
37656.90	145.00	195.00	0.85	10354.10	81.06	193.50	0.42	11958.80	55.87	196.00	0.70
40080.60	145.00	195.00	0.90	19649.10	82.94	195.20	0.77	22909.40	55.49	196.00	1.34
40125.20	144.00	196.00	0.91	15714.60	81.38	193.80	0.63	12265.20	53.64	195.00	0.75
42687.40	144.00	195.00	0.97	14604.50	80.37	195.00	0.59	13670.30	54.54	195.00	0.82
30090.80	145.00	196.00	0.67	21580.30	80.05	194.80	0.88	26325.60	53.36	197.00	1.59
31256.60	144.00	194.00	0.71	12438.40	81.40	193.50	0.50	17016.60	55.36	194.00	1.01
50169.40	147.00	196.00	1.11	12646.50	80.08	193.50	0.52	21107.90	53.92	196.00	1.27
37036.90	145.00	195.00	0.83	16227.40	80.29	194.60	0.66	11432.70	54.40	196.00	0.68
31379.40	146.00	196.00	0.70	17401.60	80.44	194.50	0.71	13661.20	53.68	196.00	0.83
44850.70	144.00	196.00	1.01	15521.30	81.29	193.20	0.63	16413.70	53.15	195.00	1.01
37533.50	145.00	194.00	0.85	16362.10	80.35	195.60	0.66	12490.30	54.32	194.00	0.75
36472.60	146.00	196.00	0.81	15438.40	80.48	194.00	0.63	14592.90	54.66	195.00	0.87
47736.30	146.00	194.00	1.07	17866.30	81.71	193.80	0.72	12376.60	53.80	195.00	0.75
34302.40	145.00	196.00	0.77	13831.90	81.86	195.10	0.55	17742.40	55.72	196.00	1.03
29117.80	146.00	196.00	0.65	12874.80	78.35	194.80	0.54	16614.70	55.18	196.00	0.98
33929.10	144.00	196.00	0.77	14537.00	79.61	194.50	0.60	18063.30	54.00	196.00	1.09
40111.40	144.00	196.00	0.90	15687.00	80.06	195.80	0.64	16629.70	54.28	194.00	1.01
36297.30	144.00	196.00	0.82	13135.90	81.07	195.00	0.53	16237.40	54.72	195.00	0.97
33827.20	144.00	194.00	0.77	11009.50	80.62	194.20	0.45	31793.30	55.01	194.00	1.90
45538.50	145.00	194.00	1.03	12244.80	81.20	193.90	0.50	24762.40	55.70	195.00	1.45

In addition, eleven figures are available in the dataset for a better appreciation of the tests. Figures 1 to 5 from the dataset show the measurements and nomenclature of the blocks. Figures 6 to 8 from dataset show the graphics of the resume, and their data is in the 3^rd^ spreadsheet. Finally, figures 9 to 11 show the mode of failure of the tested blocks.

## 2 Experimental Design, Materials and Methods

For testing the blocks with medium compressive strength, 120 blocks were randomly selected from the same batch within the same block production. First, 30 blocks were subjected to the compression test to determine their compressive strength. The other 90 blocks were separated for testing in the splitting tests. For testing the blocks with high compressive strength, 90 blocks were randomly selected from another batch of blocks. Like the first set, 30 blocks were reserved for the compression test.

**Fig. 3:**
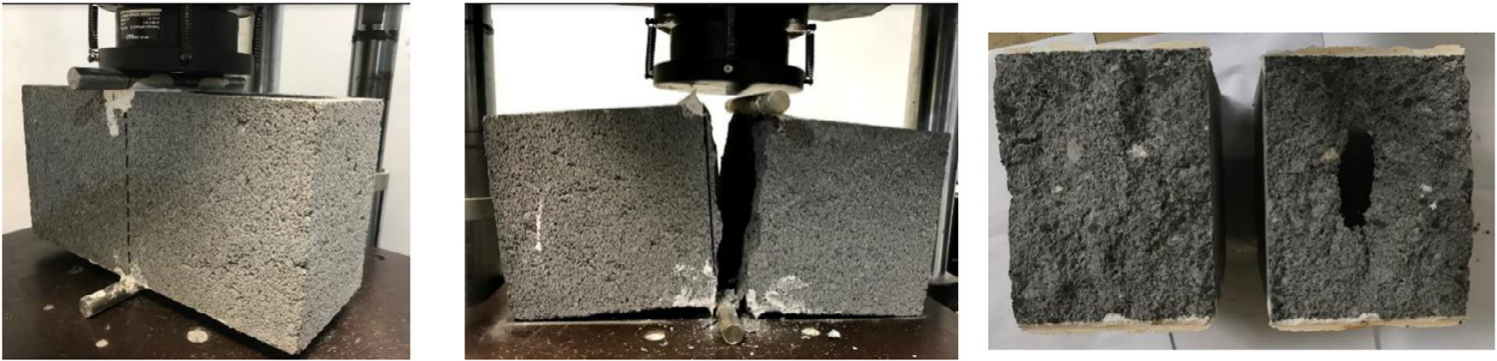
Splitting tests in the A direction.

**Fig. 4:**
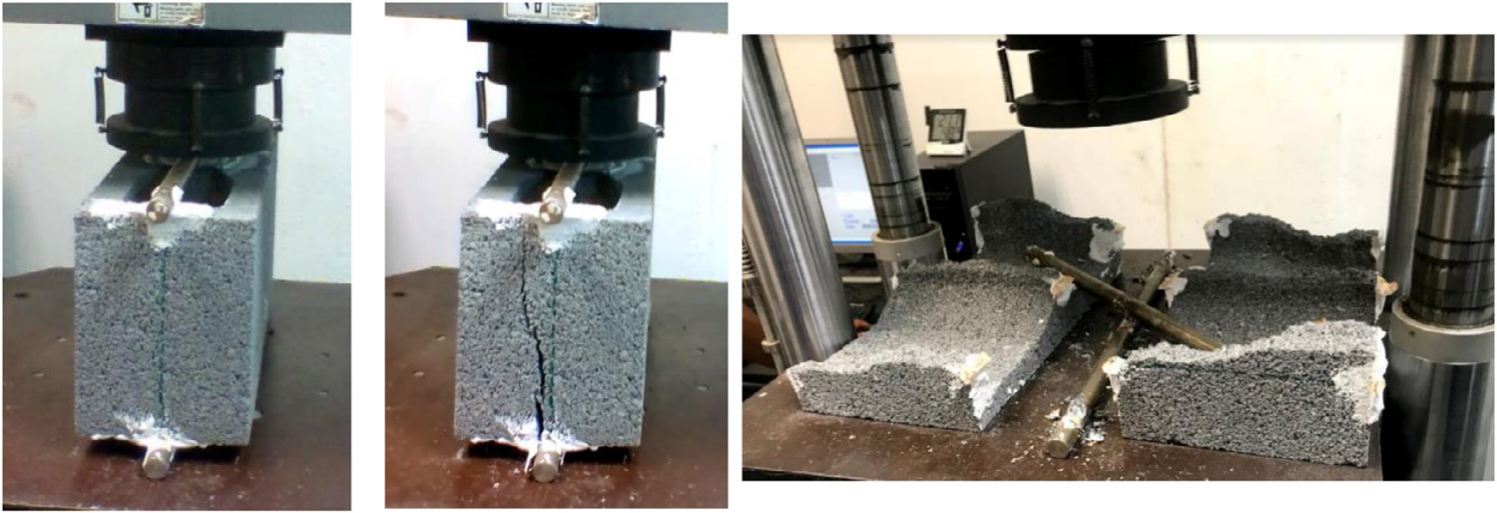
Splitting tests in the B direction.

**Fig. 5:**
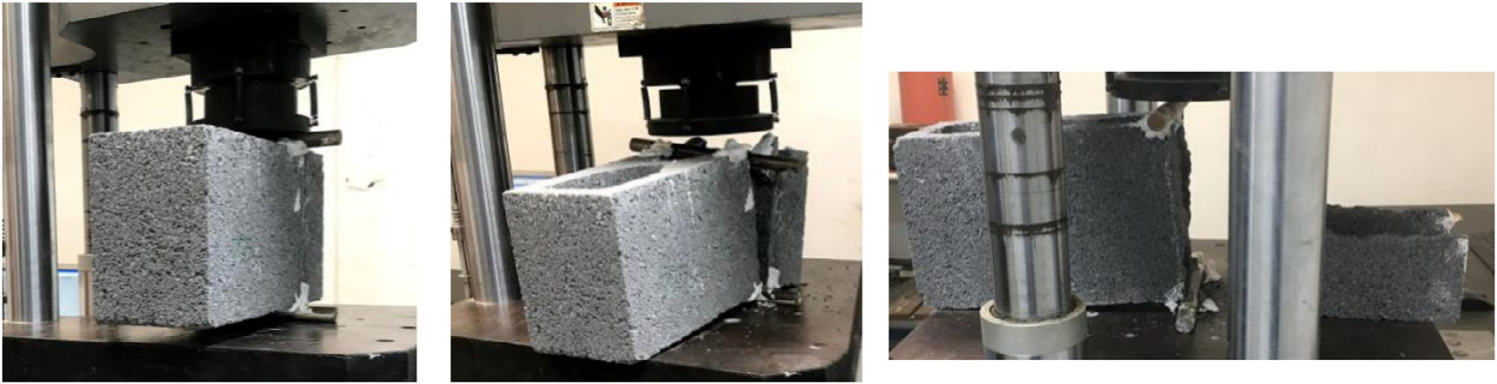
Splitting tests in the C direction.
• For blocks with high compressive strength, only 30 HCB in the A direction (Fig. 3), and 30 HCB in the B direction (Fig. 4) were tested.

In this work, the splitting test was carried out applying the ASTM – C-1006-13 standard [1]. The tests were performed in different directions:

• For blocks with medium compressive strength, 30 HCB in the A direction (Fig. 3), 30 HCB in the B direction (Fig. 4), and 30 HCB in the C direction (Fig. 5) were tested. The C direction is a variation of the ASTM test, carried out by [4,5].

## Ethics Statement

No ethical issues are associated with this work.

## CRediT Author Statement

**José Álvarez-Pérez:** Conceptualization, Methodology, Writing – Original Draft; **Milena Mesa-Lavista:** Supervision, Data Curation, Writing – original draft; **Jorge H. Chávez-Gómez:** Resources, Funding acquisition, Writing – review & editing; **Diego Cavazos-de-Lira:** Data Curation, Writing – review & editing; **Bernardo T. Terán-Torres**: Methodology, Writing – review & editing

## Declaration of Competing Interest

The authors declare that they have no known competing financial interests or personal relationships that have or could be perceived to have influenced the work reported in this article.
